# Portable Plasmonic Paper-Based Biosensor for Simple and Rapid Indirect Detection of CEACAM5 Biomarker via Metal-Enhanced Fluorescence

**DOI:** 10.3390/ijms231911982

**Published:** 2022-10-09

**Authors:** Laurentiu Susu, Adriana Vulpoi, Simion Astilean, Monica Focsan

**Affiliations:** 1 Nanobiophotonics and Laser Microspectroscopy Center, Interdisciplinary Research Institute in Bio-Nano-Sciences, Babes-Bolyai University, T. Laurian Str. 42, 400271 Cluj-Napoca, Romania; 2 Biomolecular Physics Department, Faculty of Physics, Babes-Bolyai University, M. Kogalniceanu Str. 1, 400084 Cluj-Napoca, Romania; 3 Nanostructured Materials and Bio-Nano-Interfaces Center, Interdisciplinary Research Institute on Bio-Nano-Sciences, Babes-Bolyai University, T. Laurian Str. 42, 400271 Cluj-Napoca, Romania

**Keywords:** quantum dots, plasmonic calligraphy, paper, biomarker, MEF biodetection, POC

## Abstract

Rapid, simple, and sensitive analysis of relevant proteins is crucial in many research areas, such as clinical diagnosis and biomarker detection. In particular, clinical data on cancer biomarkers show great promise in forming reliable predictions for early cancer diagnostics, although the current analytical systems are difficult to implement in regions of limited recourses. Paper-based biosensors, in particular, have recently received great interest because they meet the criteria for point-of-care (PoC) devices; the main drawbacks with these devices are the low sensitivity and efficiency in performing quantitative measurements. In this work, we design a low-cost paper-based nanosensor through plasmonic calligraphy by directly drawing individual plasmonic lines on filter paper using a ballpoint pen filled with gold nanorods (AuNR) as the colloidal ink. The plasmonic arrays were further successively coated with negatively and positively charged polyelectrolyte layers employed as dielectric spacers to promote the enhancement of the emission of carboxyl-functionalized quantum dots (QD)—previously conjugated with specific antibodies—for indirect detection of the carcinoembryonic antigen-related cell adhesion molecule 5 (CEACAM5). The efficiency, sensitivity, as well as the specificity of our portable nanosensor were validated by recording the luminescence of the QD@Ab complex when different concentrations of CEACAM5 were added dropwise onto the calligraphed plasmonic arrays.

## 1. Introduction

Cancer still remains one of the main causes of mortality worldwide, and unfortunately, many types of tumors are detected in an advanced form [1]. The survival rate for a patient is significantly improved when cancer is diagnosed at the earliest stages [2]. However, common detection methods for cancer biomarkers, such as the enzyme-linked immunosorbent assay (ELISA) and the polymerase chain reaction (PCR), remain complex, time-consuming, costly, and require specialized personnel, which, in consequence, limits their use in low-income settings and developing countries, due to lack of economic resources [3,4]. Therefore, point-of-care (PoC) tests are becoming a significant tool that offers the possibility of providing an easy, fast, and cost-effective diagnostic result in a non-laboratory environment [5]. Additionally, such settings can be scaled down into small, simple, specific, and portable devices that can screen biomarkers of interest anywhere, at any time [6]. The affordable characteristic can be mainly obtained through the use of filter paper, glass substrates, microfluidic channels, and so on; in addition, two of the most important characteristics required when designing a portable lab-on-chip (LOC) are high sensitivity and specificity when depicting the status of the disease [6,7,8,9]. As such, there is great interest in the optimization of these parameters by employing the use of plasmonic nanomaterials in designing high-throughput PoC devices, mainly gold nanoparticles (AuNP), due to their superior optical performance in the field of biosensing [10]. Specifically, these nanomaterials generate the well-known localized surface plasmon resonance (LSPR) effect when light interacts with them, which is dependent on the local refractive index surrounding the AuNP [11]. As well as enhancing the performance of existing platforms, they also provide highly sensitive and label-free detection of biomarkers. Moreover, by monitoring the optical changes with conventional spectrophotometers, the chemical and biological detection processes are simplified, and the cost is significantly reduced [12].

The majority of AuNP-based biosensors are colorimetric, and rely on the color change of the NPs on to the test strip to enable a qualitative measurement through the naked eye; however, semiquantitative results can also be obtained using readout devices, such as cameras [8]. Although promising, it still remains a challenge to obtain a highly quantitative result while also maintaining high specificity and sensitivity when detecting certain biomarkers. In this context, compared to traditional colorimetry-based nanosensors, fluorescence-based sensing and bioimaging can be a promising solution due to the fact that together they have a better detection sensitivity and improved signal-to-noise ratio [13]. Moreover, anisotropic nanoparticles, such as gold nanorods (AuNRs), are preferred when designing efficient biosensors due to their simple and scalable synthesis procedures, tunable LSPR wavelength, as well as the presence of intense electromagnetic fields at their tips, thus enabling ultrasensitive detection via metal-enhanced fluorescence (MEF) [14,15,16]. As MEF is a distance-dependent phenomenon, an important parameter in designing a biosensor is the precise control of the separation distance between the metal nanoparticle and fluorophore. This gap is usually achieved by depositing dielectric materials, such as silica, polyelectrolytes (PE), or even the biorecognition elements themselves (e.g., DNA, biotin, streptavidin), onto the predesigned nanoplatform [17,18,19]. While organic dyes are mostly used in conventional MEF biosensors as traditional signal transducers, they quench quickly and possess a very narrow Stokes shift. On the other hand, semiconductor inorganic particles, such as quantum dots (QD), represent a better candidate in practical biological applications, such as multiplexed labelling or biological immunoassays, due to their sensing capacity, high quantum yield, great photostability, larger emission–absorption spectra, and improved fluorescent properties [20,21,22,23]. Several studies have shown the use of QD as fluorescent labels in combination with nanohole arrays for substrate-enhanced luminescence in the detection of relevant biomarkers and diagnosis [24,25]. Additionally, a variety of studies employing QD as optical transducers deposited onto paper-based substrates for visible fluorescence sensing have received much interest due to their simple design, rapid analysis, and high sensitivity [26,27,28].

Therefore, in this work, we designed a low-cost, miniaturized, paper-based nanosensor through plasmonic calligraphy by directly drawing individual plasmonic lines on Whatman no. 1 filter paper, using a commercial ballpoint pen filled with colloidal AuNRs. By employing this simple approach, we are able to control and tune the plasmonic properties of our nanoplatforms by drawing separate plasmonic domains on the same paper substrate comprised of AuNRs with three different aspect ratios (denoted as AuNR680, AuNR740, AuNR860). The calligraphed plasmonic arrays were further successively coated with two layers of PE, namely, negatively charged poly (styrene sulfonate) (PSS) and positively charged poly (allylamine hydrochloride) (PAH), using the same pen-on-paper approach. This layer-by-layer (LBL) method was used to adjust the interparticle distance—acting as a dielectric spacer—leading to a fivefold luminescence intensity enhancement of QD, when the inorganic particles interacted with the nanoplatform comprised of calligraphed AuNR680, relative to the emission intensity of free QD on paper. Subsequently, for the biomarker detection, the proposed biosensing protocol involves the prior conjugation of the terminal carboxyl group (-COOH) of our QD with antibodies (QD@Ab) against carcinoembryonic antigen-related cell adhesion molecule 5 (CEACAM5), a member of the carcinoembryonic antigen (CEA) gene family. The encoded protein is used as a clinical biomarker for gastrointestinal cancers, and may promote tumor development through its role as a cell adhesion molecule [29]. The successful capture of CEACAM5 due to the strong antigen–antibody interaction was first demonstrated and evaluated by monitoring the red shift of the extinction spectrum recorded after each immobilization step. The efficiency, sensitivity, as well as the specificity of our portable plasmonic fluorescent nanosensor was validated through epifluorescence measurements, concretely by corelating the target analyte concentration to the enhanced luminescence of QD@Ab, which acts as an optical transducer, when different concentrations of CEACAM5 were added dropwise onto the plasmonic lines, allowing indirect MEF detection of the target analyte.

## 2. Results and Discussion

### 2.1. Optical and Morphological Characterization of the Designed Paper-Based Nanoplatforms

**Figure 1:**
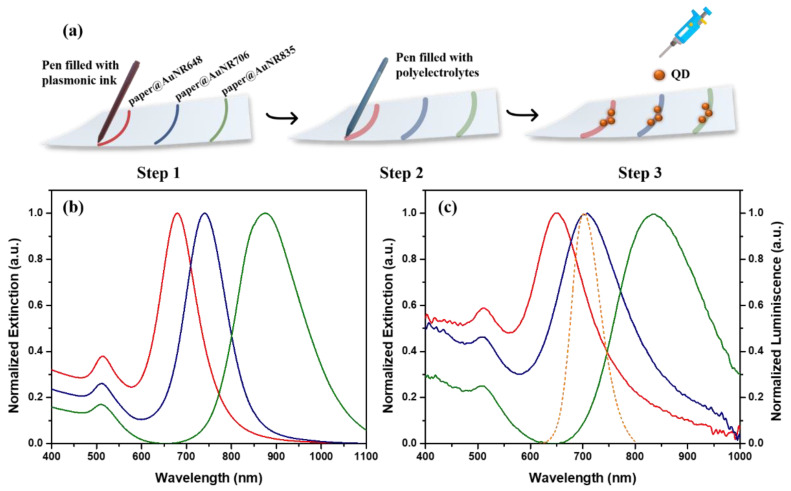
(**a**) Schematic illustration of the plasmonic calligraphy method. Step 1: fabricating the nanoplatform using a commercial pen filled with AuNRs with different aspect ratios as colloidal inks in order to draw spatially isolated plasmonic lines. Step 2: coating the plasmonic arrays with a layer of PSS, followed by a layer of PAH, using the same pen-on-paper approach. Step 3: decorating the nanoplatform with negatively charged QD. (**b**) Normalized extinction spectra of the colloidal AuNRs, with different aspect ratios: AuNR680nm—red spectrum, AuNR740nm—blue spectrum AuNR860nm—green spectrum. (**c**) Calligraphed AuNRs lines on Whatman paper (paper@AuNR648—red spectrum, paper@AuNR706—blue spectrum, paper@AuNR835—green spectrum) together with the normalized luminescence spectrum of QD with the luminescence emission band localized at 700 nm (orange dotted spectrum).

Before conducting the immobilization of the anisotropic NPs through plasmonic calligraphy onto the cellulose fibers, the colloidal AuNR inks were first characterized optically and morphologically. Figure 1b presents the normalized extinction spectra of three AuNR solutions with different aspect ratios. We can clearly see that the two characteristic LSPR contributions, namely, transversal and longitudinal bands, are present [30]. While the transverse band is located at approximately 510 nm, the longitudinal LSPR band was tuned along the spectrum, having spectral positions at 680 (red spectrum), 740 (blue spectrum), and 860 nm (green spectrum). Zeta potential measurements were then performed to determine the surface charge of our CTAB-stabilized nanostructures. The obtained value is 37 ± 0.5 mV (, which allows the easy integration of the AuNRs into the 3D porous paper matrix, consisting of interwoven α-cellulose fibers, via electrostatic interaction [31,32]. In view of designing our portable plasmonic nanosensor, schematically illustrated in Figure 1a, step 1, we employed a simple and low-cost fabrication protocol, namely, the pen-on-paper approach, to draw spatially isolated plasmonic arrays on the same paper substrate, using AuNRs with different LSPR responses. The optical response of our plasmonic lines was then analyzed by monitoring the extinction spectra of our immobilized AuNRs onto the paper fibers. As we can see in Figure 1c, no major spectral modifications, such as uncontrolled NPs aggregation or spectral broadening, were observed. The only significant difference in the optical response was the expected blue shift of the longitudinal LSPR band between 32 nm and 40 nm, which was caused by the change in the local refractive index surrounding the AuNPs from n_water_ = 1.333 to n_air_ = 1. By employing plasmonic calligraphy, we were able to obtain tunable plasmonic nanoarrays with longitudinal LSPR responses located at 648 nm (Figure 1c, red spectrum), 706 nm (Figure 1c, blue spectrum), and 835 nm (Figure 1c, green spectrum), hereby referred to as paper@AuNR648, paper@AuNR706, and paper@AuNR835, respectively. It is important to note that the AuNRs were purposely synthesized to possess longitudinal LSPR bands located in and out of resonance with the luminescence maximum of QD in order to obtain a better overview of the nanoplatform’s behavior as a possible MEF nanosensing platform. The spectral overlap between the nanoplatform paper@AuNR706 (blue spectrum) and the emission spectrum of QD located at 700 nm (using a fixed excitation wavelength at 450 nm) (orange dashed spectrum) can be clearly seen in Figure 1c.

**Figure 2:**
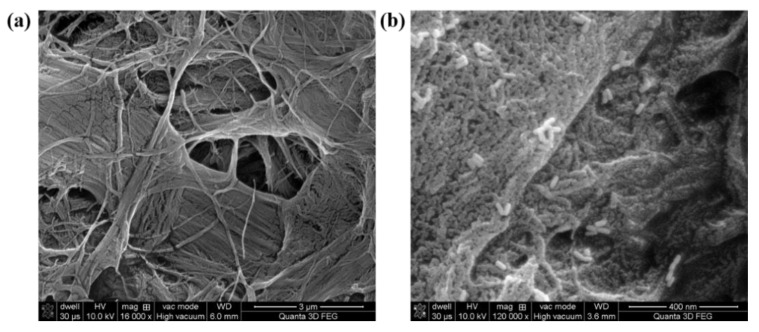
Illustrative SEM images of the bare Whatman filter paper (**a**) before and after the calligraphy step, using colloidal AuNRs as ink (**b**).

The size of the nanoparticles, as well as the morphology of the plasmonic calligraphed substrates, were investigated in order to prove the presence of AuNRs immobilized on the fiber strands of the cellulose. For exemplification, representative SEM images of the nanoplatform paper@AuNR648 were successfully recorded before (Figure 2a) and after (Figure 2b) one-line calligraphy using colloidal AuNR680 as plasmonic ink. Figure 2b illustrates an individual distribution of the AuNRs onto the paper fibers, seen as bright spots on the gray-colored paper matrix, without visible large-scale aggregation. This observation is also confirmed by the recorded LSPR spectra of the nanoplatforms, which exhibit the preserved optical properties of the AuNRs after the calligraphy process. Moreover, the dimensions of our nanostructures with the longitudinal LSPR located at 680 nm were evaluated by analyzing the corresponding SEM images, obtaining an average length of 56 nm.

**Figure 3:**
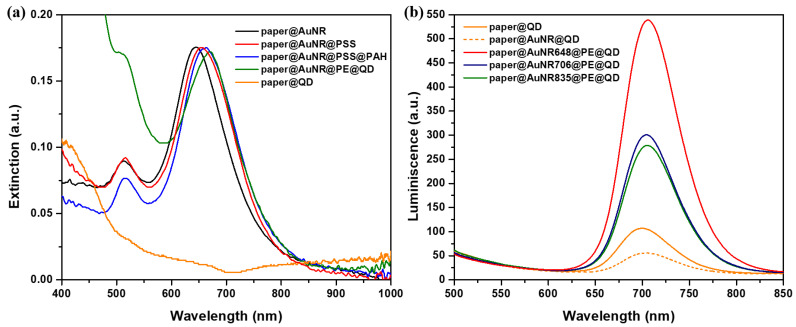
(**a**) Extinction spectra of the paper@AuNR648 nanoplatform before (black spectrum) and after being coated first with PSS (red spectrum), followed by PAH (blue spectrum), and after decorating with QD (green spectrum), together with the absorption spectra of QD on bare paper (orange spectrum), using the same concentration and experimental condition. (**b**) Luminescence spectra of paper@QD (orange spectrum), paper@AuNR@QD (orange dashed spectrum), and QD decorated on paper@AuNR@PE@648 (red spectrum), paper@AuNR@PE@706 (blue spectrum), and paper@AuNR@PE@835 (green spectrum) nanoplatforms.

In order to design an efficient and simple plasmonic substrate with advantageous capabilities in terms of enhancing the luminescence of QD, the plasmonic lines were first coated with a layer of negatively charged PSS, using the same pen-on-paper approach (see Figure 1a, step 2), followed by a second coating with a layer of positively charged PAH, to act as an interparticle spacer, since MEF is a distance-dependent phenomenon [20]. In consequence, the transversal and longitudinal LSPR bands of the immobilized AuNRs (herein, for exemplification, paper@AuNR648) undergo successive red shifts after each layer-by-layer deposition—approximately 10 to 15 nm for each layer (Figure 3a, Figure S1-red and blue spectra). Subsequently, after decorating each plasmonic array with negatively charged QD (Figure 1a, step 3), we can first see the broadening of the spectra in the 400–500 nm region as well as an additional red shift of around 10 nm of the longitudinal LSPR band (Figure 3a, green spectrum), which confirms the successful attachment of the QD to the positively charged PAH layer.

Furthermore, we investigated the effective MEF capability of our designed plasmonic nanoplatforms by comparing the luminescence of QD dropped onto plasmonic lines with the emission intensity on bare paper (paper@QD), using the same excitation wavelength. As we can see in Figure 3b, when the QD are dropped directly on to the plasmonic lines (paper@AuNR@QD), in the absence of a dielectric spacer, the luminescence of the inorganic NPs is quenched significantly (Figure 3b, orange dashed spectrum). However, when the QD are placed further away from the AuNRs, i.e., when the dielectric spacer is present to prevent quenching, all three QD-decorated paper@AuNR complexes (further denoted as paper@AuNR@PE@QD) exhibit up to a fivefold luminescence enhancement as compared to free QD on bare paper (Figure 3b, orange spectrum). Additionally, apart from the increased luminescence intensity, the band maximum of the QD red shifts after interacting with the plasmonic nanoplatforms, which can be attributed to the interaction of the QD with the immobilized AuNR@PE complex.

Of note, all the emission spectra were recorded under identical excitation and experimental conditions; for this reason, the MEF enhancement factors were calculated directly from the ratio between the emission intensities of QD grafted onto the nanoplatforms and QD on bare paper. The plasmon-enhanced fluorescence appears due to the enhanced near field of the nanoparticle, which contributes to an increase in the radiative decay rate near the plasmonic nanoparticles of the QD [33]. It is important to mention that the resulting MEF factor is significantly different for different nanoplatforms. More specifically, the nanoplatform paper@AuNR648@PE presented the best luminescence enhancement from all the samples, and in consequence, represents the best candidate of choice employed in subsequent biodetection studies. It is also important to mention that when determining the most effective position of the longitudinal LSPR band of the nanoplatform for MEF—apart from the optimal interparticle distance—we needed to take into account the LSPR shift of our nanostructures after being decorated with QD [14]. This parameter can explain the higher luminescence enhancement of the nanoplatform paper@AuNR648@PE compared to the other plasmonic arrays, which have a maximum absorption of the longitudinal LSPR band located at 670 nm.

### 2.2. Characterization of the Active QD@Ab Complex

**Figure 4:**
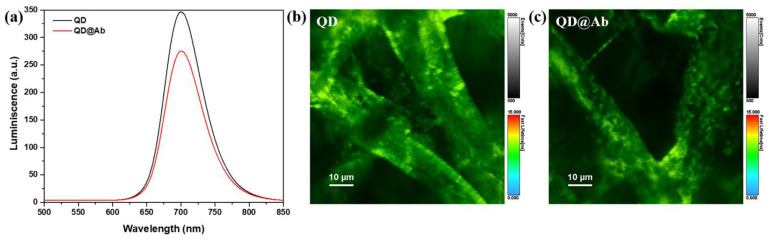
(**a**) Luminescence spectra of QD (black spectrum) and QD@Ab (red spectrum) in PBS solution (λ_excitation_ = 450 nm); Corresponding FLIM images of QD (**b**) and QD@Ab (**c**) dropped onto the paper platform (λ_excitation_ = 405 nm).

Prior to the implementation of the biosensing protocol, the QD were chemically labelled in solution via NHS/EDC chemistry with antibodies against CEACAM5 (Ab) (see experimental details in Section 3.4), and the resulting active complex was then optically characterized. The fluorescence spectra of both QD and the QD@Ab complex were recorded and compared, as can be seen in Figure 4a. The QD in PBS solution presents a maximum fluorescence intensity located at 700 nm, and after being covalently coupled to Ab, it retaines its maximum peak position.

Generally, the luminescence peak position of CdSe/ZnS core/shell QD is usually determined by the band gap of CdSe, and it is not affected by its hydrodynamic size or ligands [34]. Moreover, the fluorescence intensity of the QD@Ab complex is slightly lower than that of free QD, which may be caused by the dilution of QD in the reaction process, or due to loss in the centrifugation step [35]. Still, no shift was found in the maximum emission peak compared with that of the free QD, which suggests that the coupling of antibodies did not change the luminescence properties of the QD. Several studies have also shown that the covalent binding of antibodies to QD using NHS/EDC chemistry does not significantly alter their luminescence properties [35,36]. To gain better insight into the behavior of the QD in presence and absence of the antibodies, we investigated the changes in the QD-excited state lifetime, by acquiring and analyzing the fluorescence lifetime images of free QD (Figure 4b) and the QD@Ab (Figure 4c) complex grafted onto the paper fibers under a fixed excitation wavelength of 405 nm. The decay fitting parameters are listed in Table 1, and as can be seen from the collected data, the fluorescence lifetime of free QD on paper shows has an average value of 14 ns, which is in good agreement with the interval of 10–30 ns provided by the manufacturer’s specification data [37]. However, the average lifetime value for the QD@Ab has a slightly increased value of 15 ns, which confirms the interaction between the QD and the antibodies.

**Figure 5:**
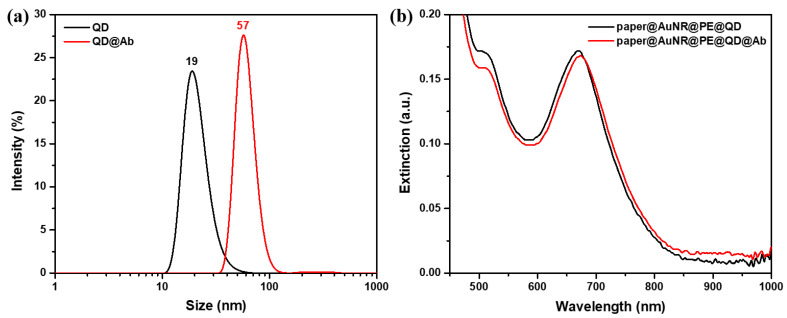
(**a**) DLS measurements of QD before (black spectrum) and after the covalent binding with Ab-CEACAM5 (red spectrum). (**b**) Comparison between the extinction spectra of paper@AuNR@PE after being decorated with free QD (black spectrum) and conjugated QD@Ab (red spectrum).

Next, to further prove the covalent binding between our luminescent nanoparticles and the antibodies against CEACAM5, we measured the size of the QD by employing DLS measurements before and after the chemical labelling with antibodies. The hydrodynamic diameter of free QD in PBS was found to be 18 ± 2 nm (Figure 5a, black spectrum), which is in good agreement with the TEM results provided by the manufacturer’s specification sheet [38]. After the chemical coupling process with antibodies, the size of QD@Ab was measured to be 57 ± 8 nm, which confirms the successful labelling; moreover, the particle size of the complex is uniformly dispersed, with no sign of aggregation (Figure 5a, red spectrum).

The coupling between QD and antibodies was further validated by collecting and comparing the extinction spectra of our nanoplasmonic platforms when interacting with QD and QD@Ab complex. As can be seen in Figure 5b, the longitudinal LSPR band corresponding to the paper@AuNR@PE@QD@Ab (Figure 5b, red spectrum) is red-shifted by 4 nm compared to the optical response of paper@AuNR@PE@QD (Figure 5b, black spectrum), which directly confirms that the QD@Ab complex interacts differently with our plasmonic nanoplatform as opposed to free QD.

### 2.3. Quantitative Detection of CEACAM5 Biomarker via Indirect MEF Sensing

As mentioned before, enhanced biosensing is an important procedure to detect low concentrations of biomarkers; however, it is challenging to design miniaturized and portable sensing nanoplatforms, as an innovative fluorimmunosensor, that allow the specific detection of antigen–antibody binding. For example, the human carcinoembryonic antigen (CEA) family has seven genes belonging to the CEACAM subgroup [39]. The CEACAM5 gene codes for the protein CEA, and is now known to be overexpressed in a majority of carcinomas, including those of the gastrointestinal tract [40]. Normal expression of this family member is restricted to the epithelial cells, and CEA is mostly found on the apical surface of the gastrointestinal epithelium. A study conducted by Topdagi et al. aimed to examine the relationship between CEA levels in the preoperative period and TNM (T, primary tumor; N, lymph node; M, distant metastasis) staging in patients with colorectal cancer [41]. They concluded that the cases with CEA ≤ 5 ng/mL were primarily in Stage III, whereas those with CEA > 5 ng/mL were predominantly in Stage IV. Another study by Bae et al. investigated the clinical usefulness of CEA expression in colorectal cancer tissue [42]. Their results show that the mean levels of preoperative serum CEA were 3.04 ng/mL, 8.34 ng/mL, 9.14 ng/mL, and 49.24 ng/mL in stages 1, 2, 3, and 4, respectively.

Moreover, there are a few studies that exploit MEF in the near-infrared region (NIR) for fluorescent PoC biosensing [43]. For instance, Jawad et al. utilized nanotriangular arrays fabricated via colloidal lithography to design an ultrasensitive immunoassay for the detection of pancreatic cancer biomarker CA 19-9, using the NIR dye DyLight800 [44]. In another study, Au nanoisland film immunoassays were used in the detection of cardiac biomarkers, cardiac troponin I (cTnI) and creatine kinase MB (CK-MB), using IRDye800 as a reporter, and the performance of the assay was validated with clinical samples [45]. Although these nanosensors have strong performance and high sensitivity, they require expensive equipment and a complex fabrication process, whereas our plasmonic calligraphy approach is much simpler and easier to implement. In consequence, the need for sensitive yet simple and inexpensive diagnostic tools have prompted the development of new strategies to enhance the fluorescence signal of diagnostic tests. In this context, to further evaluate the sensitivity of our paper-based nanoplatform fluoroimmunoassay for CEACAM5 detection, we added known concentrations of the target analyte (0–100 µg/mL) onto different plasmonic arrays using the protocol described in Section 3.4. The performance of our flexible biosensors was evaluated by monitoring the specific antigen–antibody interaction, first through the evaluation of the extinction spectra of our nanoplatforms in the presence of CEACAM5 (Figure S2). Specifically, we recorded the LSPR spectra of our plasmonic nanoplatforms when different concentrations of CEACAM5 were captured by the QD@Ab complex. As presented in Figure S2, the longitudinal LSPR red shifts when the target analyte is present, reaching a maximum detectable concentration of only 75 µL/mL of CEACAM 5.

**Figure 6:**
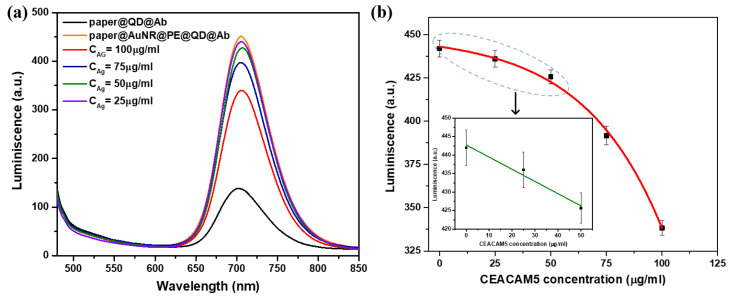
(**a**) Comparison between the luminescence spectrum of QD@Ab (black spectrum) on bare paper and on our plasmonic nanoplatforms (orange spectrum) before and after varied concentrations of CEACAM5 were added. (**b**) The calibration curve (exponential decay, red line) describing the relationship between the luminescence intensity of the QD@Ab complex and the values of the CEACAM5 concentrations; inset, linear plot (green line) of the emission intensity at CEACAM5 concentrations ranging from 0 to 50 µg/mL.

As such, we examined the effective MEF capability of our designed plasmonic nanoplatforms, since MEF is a more sensitive tool that can considerably enhance the florescence signal, by comparing the luminescence of QD@Ab complex on bare paper with the fluorescence intensity in the presence of paper@AuNR@PE, and also when different concentrations of CEACAM5 were captured by the QD@Ab, previously decorated onto the plasmonic arrays (Figure 6a).

As a first observation, the luminescence intensity of our nanoarrays decorated with QD@Ab exhibit a 3.26-fold enhancement compared to the emission of the complex on bare, which is the same behavior as previously demonstrated (Figure 6a). Secondly, the MEF factor of the QD@Ab complex appears to decrease when the target CEACAM5 concentration increases (Figure 6b, red line). This behavior can be explained by the change in the fluorescence lifetime of the QD@Ab complex, acting as an optical transducer, when the target concentration is increased, which in return decreases the quantum yield of our inorganic nanoparticles. Next, we evaluated the limit of detection (LoD) and limit of quantification (LoQ) for the detection of the CEACAM5 tumor marker by this paper@AuNR fluorescent immunoassay platform by performing a linear plot of the luminescence of the QD@Ab as a function of antigen concentration in the range 0–50 µg/mL (Figure 6b, inset). After analyzing the linear relationship from the green plot, the LoD was calculated to be 5.09 µg/mL, corresponding to a CEACAM5 concentration of 0.2 µM, while for the LoQ, we obtained an estimated value of 17.34 µg/mL. Finally, it is worth mentioning that the long-term storage of our portable paper-based plasmonic nanoplatforms was tested by recording the MEF response, concluding that they were able to detect the CEACAM5 biomarker even after three months. 

**Figure 7:**
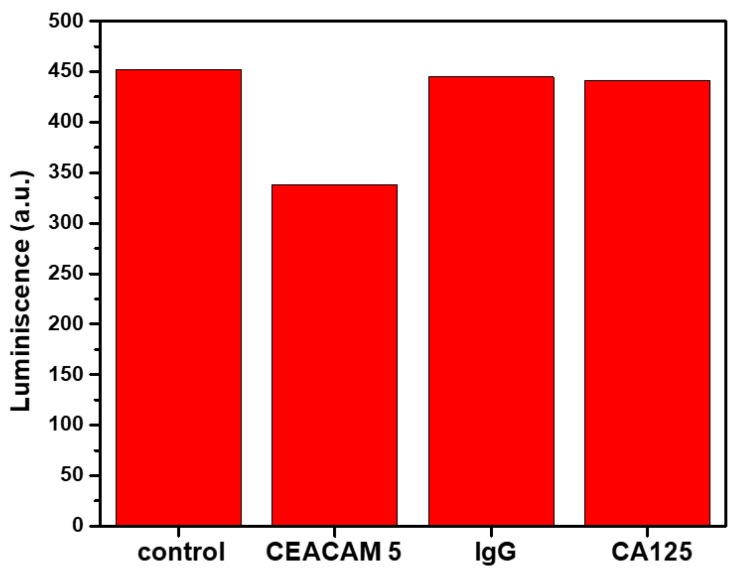
Luminescence intensities of the QD@Ab grafted onto the nanoplatform paper@AuNR648, as a control, and in the presence of CEACAM 5, human IgG and CA-125 biomarkers.

We further examined the specificity of our paperAuNR@648 plasmonic nanoplatform by monitoring the emission intensity of the QD@Ab complex in the presence of two other specimens, human IgG and CA125 in PBS (100 µg/mL). As revealed in Figure 7, we were able to determine that the presence of CEACAM5 (100 µg/mL) onto the plasmonic arrays leads to a decrease in the luminescence intensity compared to that of paper@AuNR@PE@QD@Ab (control). On the other hand, undetectable nonspecific modifications in the emission intensity could be seen for all of the other samples containing IgG or CA-125. This indicates that our fluorescent nanosensor has excellent selectivity and reliability for the detection of the CEACAM5 biomarker with minimal false-positive readouts.

Furthermore, our platform can be easily personalized and optimized to detect other types of tumor biomarkers in real life samples, such as PSA [46] or nitrated ceruloplasmin, a relevant biomarker for cardiovascular diseases or lung cancer [47]. Moreover, our proposed paper-based fluorescent biosensor could also be a strong candidate in the future for multiplex quantitative detection of tumor markers, such as AFP and CEA [48], combined biomarkers for cancer detection, such as phi [49], or simultaneous detection of free and complexed prostate-specific antigens (f-PSA and c-PSA) [50], using easy to fabricate test strips and multicolor quantum dots. Additionally, by employing QD as optical transducers, an accurate and reliable lateral flow immunoassay (LFA) can be easily developed, thus leading to an innovative and fast POC test that is able to detect cTnI, CK-MB, and Myo, which are valuable diagnostic biomarkers related to acute myocardial infarction (AMI) [51]. In this clinical context, the proposed method provides real opportunities for multiplex detection of tumor markers, and would be helpful in the early diagnosis of cancer at the site of patient care, especially in developing countries.

## 3. Materials and Methods

### 3.1. Materials

Tetrachloroauric acid (HAuCl_4_·4H_2_O, 99.99%), cetyltrimethylammonium bromide (CTAB, 96%), L-ascorbic acid (C_6_H_8_O_6_, 99%), citric acid (C_6_H_8_O_7_), sodium borohydride (NaBH_4_, 99%), silver nitrate (AgNO_3_, 99%), poly (styrene sulfonate) (PSS), poly (allylamine hydrochloride) (PAH), phosphate-buffered saline (PBS), bovine serum albumin (BSA), N-hydroxysuccinimide (NHS,98%), N-(3-Dimethylaminopropyl)-N’-ethyl carbodiimide hydrochloride (EDC), IgG from human serum (IgG, ≥ 95%), and Whatman^®^ Grade 1 Qualitative Filter Papers were purchased from Sigma-Aldrich, St. Louis, MO, USA. ITK Core/shell CdSe/ZnS QuantumDots705 functionalized with carboxyl terminal groups were purchased from Thermo Fisher Scientific, Waltham, MA, USA. Polyclonal Anti-CEACAM5 affinity isolated antibody produced in rabbits (RRID: AB_1009029), PrEST Antigen CEACAM5 (molecular weight ~25 kDa), and PrEST Antigen MUC16 (molecular weight ~25 kDa), also known as ovarian cancer-related tumor marker CA125, were purchased from Atlas Antibodies. All chemicals were used without further purification. Ultrapure water (resistivity ~18.2 MΩ) was used as a solvent throughout the experiments.

### 3.2. Colloidal Gold Nanorods Synthesis

AuNRs of three different aspect ratios (AuNRs680, AnNR740, AuNRs873) were synthesized using the two-step seed-mediated approach proposed by Nikoobakht et al. with some minor modifications [52]. First, the seed solution was prepared by mixing 0.4 M CTAB with 1 mM HAuCl_4_ and 10 mM of freshly prepared ice-cold NaBH_4_ reducing agent solution under 2 min of vigorous stirring. Then, 12 to 16 µL of the obtained gold seeds were added to a growth solution, consisting of 0.2 M CTAB, 1 mM HAuCl_4_, AgNO_3_, and 78.8 mM ascorbic acid as the reducing agent added during mild stirring. The mixture was left undisturbed at room temperature until it stabilized. The resulting gold nanoparticles solutions were purified twice by centrifugation at 14,000 rpm for 15 min followed by redispersion in ultrapure water for further use.

### 3.3. Preparation of the Plasmonic Nanoplatforms

Commercially available ballpoint Schneider pens with empty refillable cartridges, were bought from a local library store. Concentrated solutions of AuNRs were injected into the pen cartridges, which were then used to draw three 1 mm wide plasmonic lines, onto a Whatman no. 1 paper strip (Figure 1a, step 1). Multiple lines were retraced in the same position to increase the optical density of the NPs, thus optimizing the efficiency of the nanoplatform for further experiments until our plasmonic substrate was fully optimized for sensing experiments. After the immobilization of the NPs on to the paper substrate, the plasmonic arrays were firstly coated with a negatively charged layer of PSS using the same pen-on-paper approach, followed by another layer of positively charged PAH (Figure 1a, step 2). After each tracing, the nanoplatforms were left to dry at room temperature to avoid damaging the cellulose fibers. Finally, 5 µL of negatively charged QD 0.1 µM (−45.9 mV, zeta deviation = 0.5 at pH 8) were added dropwise onto the plasmonic arrays, which were positively charged due to the PAH layer previously added (Figure 1a, step 3)

### 3.4. Biosensing Protocol

**Scheme 1:**
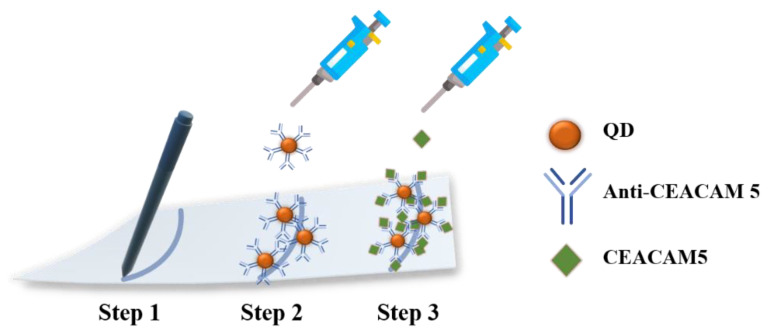
Schematic illustration of the proposed biosensing protocol. Step 1: successively coating the plasmonic arrays with two layers of PE. Step 2: decorating the nanoplatform with QD@Ab as recognition elements. Step 3: adding 5 µL of CEACAM5 biomarker target analyte (captured via specific antigen–antibody interaction).

The proposed biosensing protocol, schematically illustrated in Scheme 1, implies the conjugation of the QD with antibodies against CEACAM5 (Ab) using standard NHS/EDC chemistry [53]. To begin, 100 µL of 0.2 µM QD was gently mixed with 50 µL NHS and 50 µL EDC (with a molar ratio of NHS/EDC = 4:1), and left to react for 15 min in order to activate the –COOH groups of the core/shell nanoparticles. Afterwards, 100 µL of 1 mg/mL Ab-CECAM5 was added to the mixture and incubated for 2 h at 4 °C. Then, a 100 kDa ultrafiltration membrane was used to centrifuge the mixture at 6000 RPM at room temperature for 10 min to remove unreacted products; the resulting pellet was resuspended in PBS for further use. After the purification step, 5 µL of QD@Ab complex was added dropwise onto the plasmonic substrate previously coated with PE and left to dry at room temperature for 10 min. The nonspecific binding sites were blocked using a 1 mg/mL BSA in PBS solution and finally 5 µL of different concentrations of CEACAM5 in PBS were added on the nanoplatform and left for 20 min. Of note, the experiments were performed multiple times using three of our designed nanoplatforms for the detection of each CEACAM5 concentration.

In order to calculate LoD and LoQ values, the model proposed by Shrivastava et al. [54] was used, which is expressed as:

LoD = 3 × S_error_/Slope

LoQ = 10 × S_error_/Slope

where S_error_ represents the standard deviation error.

### 3.5. Characterization Techniques

The UV–vis–NIR extinction spectra of the colloidal AuNRs were measured using a Jasco V-670 spectrophotometer with a 2 nm bandwidth and 1 nm spectral resolution.

The zeta potential of the anisotropic NPs and QD was analyzed using a Nano ZS90 Zetasizer analyzer from Malvern Instruments equipped with a He-Ne laser (633 nm, 5 mW) in a 90° configuration, at 25 °C. Particle hydrodynamic diameter and size distribution of QD and QD@Ab were determined by dynamic light scattering (DLS) measurements, using the same Zetasizer NanoZS90 equipment from Malvern Instruments.

The optical properties of the designed paper-based nanoplatforms were collected using a portable Ocean Optics USB 4000 optical UV-Vis spectrophotometer, with a spectral resolution of 0.2 nm, coupled to a ZEISS Axio Observer Z1 inverted microscope with a 10× ZEISS objective (NA = 0.45) through an optical fiber with a core diameter of 600 μm. The extinction spectra were recorded in absorption mode, in the optical range 400–1000 nm using five accumulations and a 50-millisecond integration time.

Scanning electron microscopy (SEM) was used to investigate the size, morphology as well as the uniform distribution of the AuNRs onto the cellulose strands. Surface SEM observations were carried out using a FEI Quanta 3D FEG dual beam scanning electron microscope operating at an accelerating voltage of 10 kV. The plasmonic nanoplatforms were sputtered using a Q150R ES automatic Sputter Coater, in an argon atmosphere, with 5 nm gold layer prior to the SEM investigation in order to inhibit charging, reduce thermal damage and improve the secondary electron signal required for topographic examination in the SEM.

Luminescence spectra were recorded using a Jasco FP-6500 spectrofluorometer with a 1 nm spectral resolution, equipped with a Xenon lamp as excitation source coupled to an epifluorescence module (EFA 383 module). Fluorescence spectra were collected in the wavelength range of 500–850 using a fixed excitation wavelength at 450 nm. We chose to use the 450 nm as excitation wavelength because of the greater light absorption of the QD in the blue to violet spectral region.

Fluorescence lifetime measurements were performed on a PicoQuant MicroTime 200 time-resolved confocal fluorescence microscope system (PicoQuant GmbH, Berlin, Germany) based on an inverted microscope (IX 71, Olympus). The FLIM images of the QD adsorbed onto paper or QD@Ab grafted onto the paper substrate were performed using a 405-laser diode (LDH-D-C-405). Time-resolved fluorescence decay curves were recorded and analyzed using the SymPhoTime software (version 1.6) provided by PicoQuant. The fluorescence lifetimes were obtained through nonlinear iterative deconvolution algorithm. The instrument response function (IRF) was recorded from the laser light back scattered from plain cover glass working in similar experimental conditions. The quality of the fits was judged by analyzing the chi-square (χ^2^) values and the distribution of the residuals.

## 4. Conclusions

In summary, we have designed a sensitive, quick, efficient, and affordable paper-based immunoassay nanoplatform for quantitative detection via MEF of CEACAM5 tumor marker. Successful immobilization through plasmonic calligraphy and the preserved optical properties of the AuNRs onto the paper fibers was confirmed by monitoring the extinction spectra and via SEM images. The three spatially isolated plasmonic arrays comprised of AuNRs with different aspect ratios were further successively coated with negatively and positively charged PE, thus creating an optimal gap between the anisotropic nanoparticles and inorganic QD for MEF to occur. By playing the role of a dielectric spacer for keeping the QD at a favorable distance from the AuNRs, our nanoplatform paper@AuNR648 triggers a fivefold enhancement of the luminescence of QD compared to their emission on bare paper. Finally, the MEF sensing potential of our plasmonic fluorescent nanosensor paper@AuNR648 was confirmed by indirectly monitoring the enhanced luminescence intensity of the active QD@Ab complex in the presence of different concentration of CEACAM5, using epifluorescence measurements, achieving an LoD of 0.2 µM of tumor biomarker. We assume that these enhancement factors can be improved by precise nanoscale engineering as well as by controlling the fluorescence interaction and distance between the NPs and the QD, thus achieving an even lower LoD. The specificity of our fluorescent nanosensor was further assessed through the undetectable nonspecific absorption of several other biomarkers. Moreover, we expect our inexpensive plasmonic florescent nanosensor, fabricated using the pen-on-paper approach, to have broad usage for early disease monitoring, and could be a strong candidate in the future for promoting multiplex detection of clinical biomarkers.

## Figures and Tables

**Table 1:** Decay Parameters of free QD and QD conjugated with antibodies on paper.

Excitation (nm)	Sample	τ_1_ (ns)	τ_2_ (ns)	⟨τ⟩ (ns)	χ^2^
405	QD	15 ± 0.29	1.15 ± 0.01	14 ± 0.28	1.06
	QD@Ab	16 ± 0.24	0.99 ± 0.01	15 ± 0.23	1.13

Note: τ_1_ and τ_1_ are the lifetimes of the first and second component; ⟨τ⟩ is the intensity-weighted average lifetime. χ^2^ measures the goodness of fit (1 corresponds to a perfect fit).

## Data Availability

Not applicable.

## Supplementary Materials

The following supporting information can be downloaded at: https://www.mdpi.com/article/10.3390/ijms231911982/s1.
