# Supplementary material for: Portable Plasmonic Paper-Based Biosensor for Simple and Rapid Indirect Detection of CEACAM5 Biomarker via Metal-Enhanced Fluorescence
Source: Int J Mol Sci. 2022 Oct 9;23(19):11982. doi: 10.3390/ijms231911982 (PMC9569726; doi:10.3390/ijms231911982)
Supplement: Supplementary file 1 [file ijms-23-11982-s001.zip › ijms-1949492-supplementary.pdf]

Portable plasmonic paper-based biosensor for simple and rapid indirect detection of CEACAM5 biomarker via Metal Enhanced Fluorescence

Laurentiu Susu[1,2], Adriana Vulpoi[3], Simion Astilean[1,2], Monica Focsan[1,*]

1 Nanobiophotonics and Laser Microspectroscopy Center, Interdisciplinary Research Institute in Bio-Nano-Sciences, Babes-Bolyai University, T. Laurian Str. 42, 400271, Cluj-Napoca, Romania

2 Biomolecular Physics Department, Faculty of Physics, Babes-Bolyai University, M. Kogalniceanu Str. 1, 400084, Cluj-Napoca, Romania

3 Nanostructured Materials and Bio-Nano-Interfaces Center, Interdisciplinary Research Institute on Bio-Nano-Sciences, Babes-Bolyai University, T. Laurian Str. 42, 400271, Cluj-Napoca, Romania

* Correspondence: monica.iosin@ubbcluj.ro

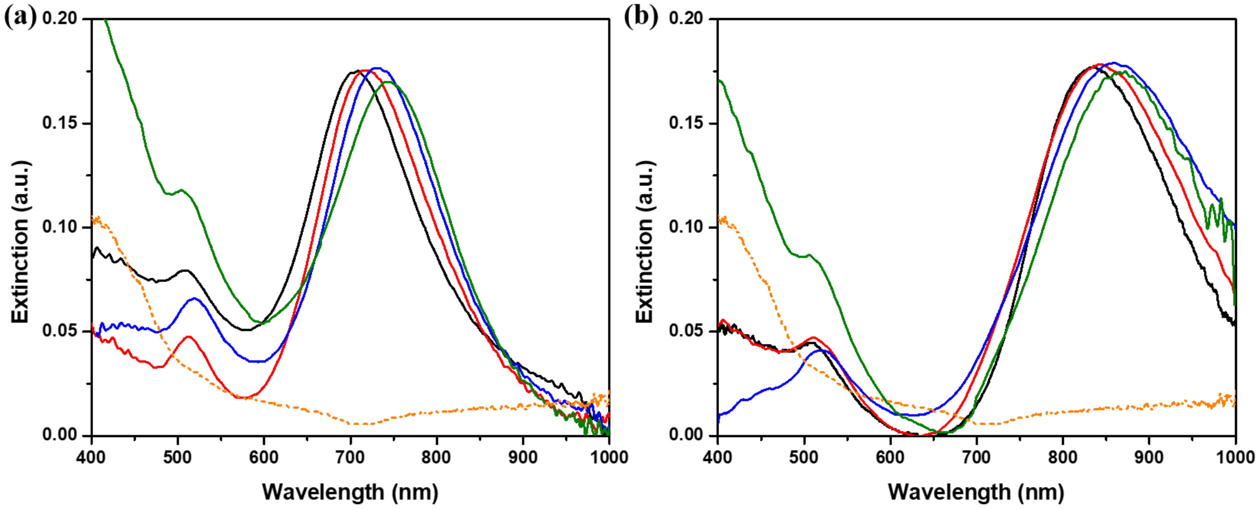

Figure S1. Extinction spectra of **(a)** paper@AuNR706 and **(b)** paper@AuNR835 nanoplatforms before (black spectrum) and after being coated first with PSS (red spectrum), followed by PAH (blue spectrum) and after decorating with QD (green spectrum) together with the absorption spectra of QD on bare paper (orange spectrum).

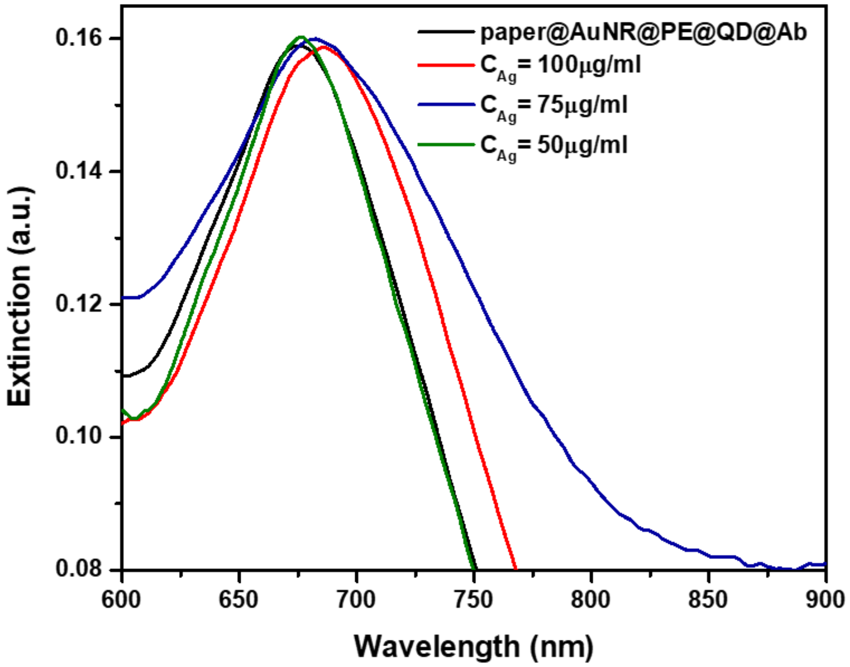

Figure S2. Extinction spectra of the paper@AuNR648 nanoplatform before (black spectrum) and after different concentrations of CEACAM5 were captured by the QD@Ab complex.
